# Differentiating scleroderma renal crisis from other causes of thrombotic microangiopathy in a postpartum patient

**DOI:** 10.5414/CN107465

**Published:** 2012-05-14

**Authors:** Muaz Abudiab, Megan L. Krause, Mary E. Fidler, Karl A. Nath, Suzanne M. Norby

**Affiliations:** 1 Department of Internal Medicine,; 2 Department of Laboratory Medicine and Pathology, and; 3 Division of Nephrology and Hypertension, Department of Internal Medicine, Mayo Clinic, Rochester, MN, USA

**Keywords:** thrombotic microangiopathy, scleroderma, postpartum, mixed connective tissue disease

## Abstract

Thrombotic thrombocytopenic purpura (TTP), hemolytic uremic syndrome (HUS), and scleroderma renal crisis (SRC) all present with features of thrombotic microangiopathy. Distinguishing among these entities is critical, however, as treatments differ and may be mutually exclusive. We describe the case of a 25-year-old woman with an undefined mixed connective tissue disease who presented 6 weeks post-partum with fever, transient aphasia, thrombocytopenia, hemolytic anemia, and acute kidney injury eventually requiring initiation of hemodialysis. Renal biopsy revealed thrombotic microangiopathy. Renal function did not improve despite immediate initiation of plasma exchange, and an angiotensin-converting enzyme (ACE) inhibitor was initiated following discontinuation of plasma exchange. At last follow up, she remained dialysis dependent. Due to the myriad causes of thrombotic microangiopathy and potential for diagnostic uncertainty, the patient’s response to therapy should be closely monitored and used to guide modification of therapy.

## Introduction 

Thrombotic microangiopathies are a diverse group of disorders that clinically present in a similar manner. Thrombotic thrombocytopenic purpura (TTP), first described in 1924, is a rare but potentially lethal condition [1]. The diagnosis is made clinically and is classically characterized by a pentad of thrombocytopenia, microangiopathic hemolytic anemia, transient neurological symptoms, renal dysfunction, and fever. Although the introduction of therapeutic plasma exchange in the 1970’s has improved survival rates, mortality remains 10 – 20% [2]. In 1998, the presence of large von Willebrand factor multimers in idiopathic TTP was ascribed to deficiency of the von Willebrand factor-cleaving protease (ADAMTS13) enzyme [3]. Hemolytic uremic syndrome (HUS) is most often associated with infection by *Escherichia coli* 0157:H7, which produces a verotoxin that mediates endothelial damage. Increasingly, atypical forms of HUS (aHUS) are recognized to result from disorders of complement regulation, including antibodies to or mutations in the genes encoding complement factors B, H, and I as well as cell surface marker CD46, complement component 3 (C3), and thrombomodulin [4]. Pathological specimens from target organs (kidneys, cerebral vasculature, and skin) reveal thrombotic microangiopathy (TMA). This finding is seen in a number of processes, including malignant hypertension, tumor cell embolism, paroxysmal nocturnal hemoglobinuria, humoral rejection in transplanted organs, antiphospholipid antibody syndrome, postpartum state, drug-mediated endothelial damage, and scleroderma renal crisis (SRC) [5]. SRC results in new onset of significant systemic hypertension and renal dysfunction [6]. Early treatment with angiotensin-converting enzyme (ACE) inhibitors has reduced 12-month mortality of SRC to less than 15% [7]. Distinguishing other causes of TMA from SRC can be difficult. However, simultaneous therapy with plasma exchange and an ACE inhibitor is contraindicated due to the potential for developing a bradykinin-mediated reaction resulting in severe hypotension [8].Existing literature discussing the diagnostic difficulty in differentiating other causes of TMA, such as post-partum TTP, from SRC is scarce [9]. Herein, we report the 6^th^ case of scleroderma renal crisis (with overlap features of TTP) in mixed connective tissue disease (MCTD) [10, 11, 12, 13, 14], but only the second in the postpartum period. 

## Case report 

**Figure 1.**
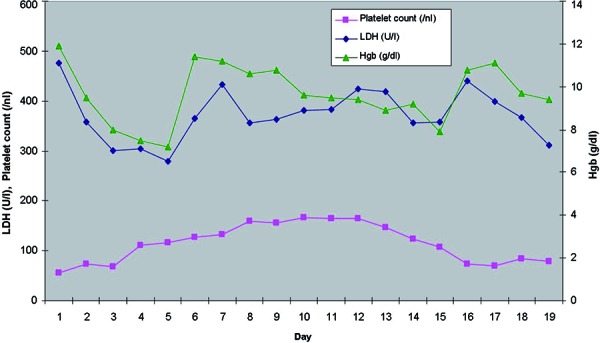
Laboratory data during clinical course.

**Figure 2.**
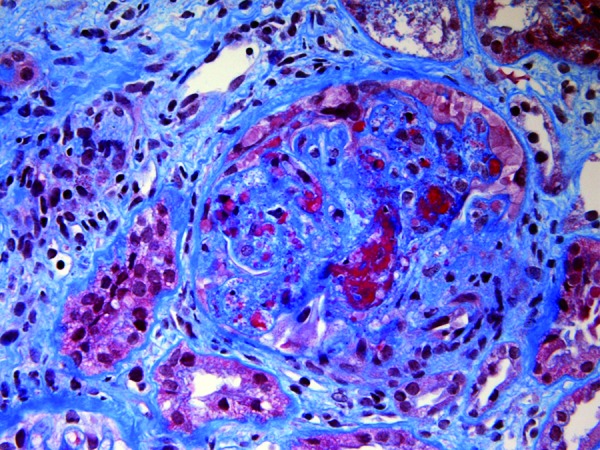
Masson’s trichrome stain (× 40) showing glomerulus with segmental thrombosis and mesangiolysis.

**Figure 3. Figure3:**
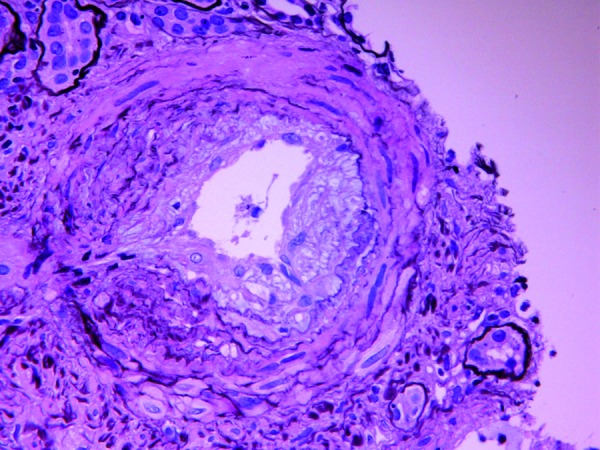
Jones’ methenamine silver stain (× 40) showing renal artery with prominent myxoid intimal thickening

A 25-year-old woman with mixed connective tissue disease (MCTD) presented to another institution 6 weeks post-partum with low-grade fever, generalized malaise, nausea, and dyspnea on exertion. Two transient episodes of aphasia with bilateral lower facial weakness were also reported. She had recently been restarted on prednisone 40 mg daily after developing dyspnea and pleuritis, thought to be an exacerbation of MCTD, which was characterized by positive antinuclear antibodies, anti-Smith antibody, and anti-ribonucleoprotein antibody. Anti-topoisomerase antibodies (anti-Scl-70) and anti-centromere antibodies were negative. Upon presentation, she had uncontrolled hypertension, periorbital edema, and livedo reticularis. On admission to our institution, she was noted to have non-oliguric acute kidney injury with serum creatinine 4.3 mg/dl, nephrotic range proteinuria, normocytic anemia with hemoglobin 10.4 g/dl, and thrombocytopenia with a platelet count of 63 × 10^9^/l. As illustrated in Figure 1, the anemia and thrombocytopenia were progressive, and lactate dehydrogenase (LDH) was elevated. Haptoglobin level was low at 23 mg/dl. Complement levels were normal. Rare schistocytes were seen on peripheral blood smear, consistent with hemolysis. A renal biopsy was performed, revealing active and chronic thrombotic microangiopathy (Figure 2) by light microscopy processed urgently. In view of recent neurologic symptoms, acute kidney injury, thrombocytopenia, microangiopathic hemolytic anemia, and history of low grade fever, a presumptive diagnosis of TTP was made. Plasma exchange was initiated emergently, and prednisone was increased to 80 mg daily with subsequent improvement in platelet count and LDH. In addition, hemodialysis was initiated for worsening kidney injury.[Fig Figure3]

After four sessions of plasma exchange, however, LDH began to rise despite normalization of haptoglobin and resolution of schistocytes on peripheral smear (Figure 1). The ADAMTS13 level, sent prior to initiation of plasma exchange, returned as normal. As the patient’s course evolved, the concern was raised for a possible scleroderma process rather than a manifestation of chronic TTP. Plasma exchange was discontinued, and the patient was initiated on captopril for presumed scleroderma renal crisis. Her previous antihypertensive regimen had consisted of amlodipine, metoprolol, and clonidine. While the microangiopathic hemolytic anemia and thrombocytopenia resolved, renal function did not improve, and she remained dialysis dependent 5 months after the initial diagnosis. 

## Discussion 

This case illustrates the diagnostic difficulty in differentiating among the various causes of TMA in a post-partum female with a history of MCTD. Table 1 compares clinical characteristics of various causes of peripartum TMA. Castella et al. reported that among patients with TTP, 10 – 25% were pregnant or in the postpartum period [15, 16]. The pentad classically seen with TTP, which results from microvascular platelet clumping [9], can also arise with SRC. Prompt initiation of therapy (plasmapheresis) for the former is mutually exclusive with that (ACE inhibition) of the latter given the threat of a bradykinin-mediated reaction [8]. Several strategies may be used to distinguish between the two conditions. First, while the pathophysiology of TTP is typically mediated by deficiency of von Willebrand factor cleaving protease (ADAMTS13), SRC results from endothelial injury and intimal proliferation [17]. Although ADAMTS13 activity has not been studied in SRC, the assay has good specificity for “idiopathic” TTP (91% in one large case series) [18]. Consequently, severe ADAMTS 13 deficiency (< 5%) may be used to help confirm the clinical diagnosis of idiopathic TTP. High suspicion for TTP typically mandates rapid initiation of treatment without the luxury of an ADAMTS13 level given the prolonged turnaround time, but the assay can be incorporated later in the clinical course. While considered by some to be entities along the same spectrum as TTP, some forms of aHUS are now known to be due to dysregulation at various points in the complement system [4] with testing for these disorders not widely available clinically. This further complicates arrival at an accurate and timely diagnosis of a patient presenting with TMA. 

Second, the diagnosis of SRC needs to be strongly considered in patients with systemic sclerosis since 25% of that subset of patients develops SRC compared to 1% of those with the limited cutaneous form of scleroderma [19]. While our patient demonstrated no cutaneous evidence of scleroderma, including a skin biopsy, systemic sclerosis sine scleroderma was a consideration. This is an entity associated with undifferentiated connective tissue disease in which organ involvement of scleroderma is witnessed without skin involvement [20]. In these patients, SRC with poor, and even fatal, outcomes can occur [10, 15, 21]. Although it is well-established that connective tissue disorders may be exacerbated by pregnancy, it is unclear if rates of SRC are increased in pregnant women [22]. Another consideration in this case is the use of high-dose corticosteroids, which are associated with SRC [23]. While it is reasonable to implicate pregnancy as a potential precipitant given the timing of presentation, steroid exposure may have been an important risk factor. Additionally, worsening renal function was observed prior to increase of steroid dose. 

Finally, response to treatment must be critically monitored. The only established treatment of SRC is ACE inhibitor therapy [24] while early initiation of plasma exchange in other causes of TMA is often lifesaving [9]. Due to the possibility of developing severe hypotension, administration of ACE inhibitors is contraindicated in patients undergoing plasma exchange. It is recommended that ACE inhibitor therapy be discontinued 24 – 72 hours prior to initiation of plasma exchange, depending on the duration of action of the ACE inhibitor [8]. Failure of plasma exchange for suspected TTP or aHUS should prompt reconsideration of alternate diagnoses, such as SRC, and the treatment plan should be adjusted as indicated. 

**Table 1:** Comparison of common clinical characteristics of causes of peripartum TMA [17, 25, 26, 27, 28, 29, 30, 31, 32, 33, 34].

	TTP	“Typical” HUS	Atypical HUS	Pre-eclampsia	HELLP	SRC	APS
Clinical presentation	Fever, HTN, neurologic symptoms, bleeding, purpuric rash	Abdominal pain, bloody diarrhea due to verotoxin-production; neurologic symptoms possible	None or non-specific prodrome with malaise, fatigue, upper respiratory symptoms	HTN, nausea, vomiting, abnormal vision	Abdominal pain, headache, malaise, nausea, vomiting, HTN	Dyspnea, altered mentation, HTN	HTN, arterial and venous thromboses, fetal demise
Typical laboratory findings	MAHA, ↓platelets, AKI, proteinuria, hematuria	MAHA, ↓platelets, AKI, proteinuria, hematuria	MAHA, ↓platelets, AKI, proteinuria, hematuria, ↓complement	Proteinuria, hyperuricemia	MAHA, ↑AST, ↓platelets	MAHA, AKI, proteinuria, hematuria	MAHA, ↓platelets, AKI, proteinuria, hematuria, antiphospholipid antibodies
Other possible laboratory findings	Low level of ADAMTS13	Stool culture positive for verotoxin-producing *E. coli*	Abnormalities of complement regulatory proteins (Factors B, H, and I, MCP, C3, thrombomodulin)	MAHA, AKI, and ↑AST may occur in severe pre-eclampsia	AKI	Autoantibodies suggestive of scleroderma	Autoantibodies suggestive of SLE
Occurrence and timing related to pregnancy	Rare; generally < 23 – 26 weeks gestation	Rare; post-partum	Rare; post-partum	> 20 weeks gestation; occasionally postpartum	> 20 weeks gestation; occasionally postpartum	Unclear; > 24 weeks gestation when observed	1/3 of cases reported during pregnancy or postpartum period
Treatment	Plasma exchange, steroids, rituximab	Supportive	Plasma exchange, eculizumab	Anti-HTN therapy; when severe, magnesium sulfate and delivery	Delivery	ACE-inhibitor therapy	Anticoagulation, plasma exchange
Renal prognosis	ESRD is rare (0 – 6%)	CKD in 5 – 25%	ESRD in 20 – 60%	Low risk of ESRD (~ 8%)	ESRD is rare (0 – 2%)	ESRD in 20% treated with ACE-inhibitor	ESRD is rare (few case reports)

TMA = thrombotic microangiopathy; TTP = thrombotic thrombocytopenic purpura; HUS = hemolytic uremic syndrome; SRC = scleroderma renal crisis; APS = antiphospholipid antibody syndrome; HELLP = hemolysis, elevated liver enzymes, low platelets; HTN = hypertension; MAHA = microangiopathic hemolytic anemia; AKI = acute kidney injury; AST = aspartate aminotransferase; ADAMTS = a disintegrin-like and metalloprotease with thrombospondin type 1 motif; MCP = membrane cofactor protein; SLE = systemic lupus erythematosus; ACE = angiotensin converting enzyme; ESRD = end-stage renal disease; CKD = chronic kidney disease.
